# Impulsivity, Substance Abuse, and Family/Friends History of Suicide Attempts in University Students With and Without Suicidal Ideation

**Published:** 2011

**Authors:** Majid Ghaffari, Ahmad Ahmadi, Mohammad Reza Abedi, Maryam Fatehizade, Iran Baghban

**Affiliations:** 1 Department of counseling, University of Isfahan, Isfahan, Iran.

**Keywords:** Impulsivity, Substance abuse, Suicide ideation, Family

## Abstract

**Objective:** Impulsivity appears to play an important role in suicidal behavior. The aim of this cross-sectional study was to compare the impulsivity, substance abuse, and family/friends history of suicide attempt between suicide-ideated and non suicide-ideated university students.

**Methods:** The research population consisted of all the students of the University of Isfahan in the academic year of 2009-2010. Three hundred and forty students (136 boys and 204 girls) were selected randomly through cluster sampling, of whom 53 participants were suicide-ideated and the rest were non suicide-ideated. The instruments used in this study were the 11^th^ version of Barratt Impulsivity Scale, Suicide Ideation Questionnaire, and the demographic questionnaire. Descriptive statistics and multivariate analysis of variance were used to examine hypothesis.

**Results:** There was a significant difference between suicide ideated and non-suicide ideated subjects in impulsivity (F=3.83, p< 0.001). Accordingly, significant differences were observed between two groups in attentional (F=8.12, p< 0.005), motor (F=7.67, p< 0.006), and non-planning (F=4.60, p< 0.033) impulsiveness. The results showed a higher incidence of substance abuse, and family/friends suicide attempt among suicide-ideated compared with non suicide-ideated students.

**Conclusion:** A higher level of impulsivity is probably related to various indices of suicidal behavior. Substance abuse is probably associated with suicidal behavior and this association may involve an interaction with impulsivity. This study provides an initial evidence of familial linkages of suicide ideation and suggests that the loss of an important person in life would be an important predictor of suicide ideation in university students.

## Introduction

Ver the last few decades, suicide among the young has emerged as a major global public health problem. The latest mean worldwide annual rates of suicide per 100,000 inhabitants were 0.5 for females and 0.9 for males among 5–14-year-olds, and 12.0 for females and 14.2 for males among 15–24-year-olds (1). The suicide rate for people between ages 15 and 24 has shown a dramatic increase in the past 60 years, with rates doubling for females and quadrupling for males, which indicates the importance of studying suicidal behavior in this developmental group (2). Studies examining common correlates of suicidal behavior consistently identify impulsivity as an important factor in suicide risk, where higher levels of impulsive personality characteristics are related to various indices of suicidal behavior including suicidal ideation and suicide attempts (3,4). Impulsivity is usually conceptualized as the inability to resist a drive or stimulus, or a behavior that occurs without reflection or consideration for the consequences of such behavior (5). Dysregulation of emotion, impulsivity and aggressiveness might be thought of as problems in the internalization or externalization of certain emotions. The challenges of adolescence and young adulthood may aggravate internalizing or externalizing problems, resulting in serious deregulation in emotional control in anger-provoking situations. Impulsiveness and aggressiveness traits in adolescents and young adults are related to psychosocial adjustment problems, and even suicide risk (6-9). Individuals, who complete or attempt suicide, show impulsiveness and aggressiveness more frequently than demographically similar non-suicidal individuals, even when they have similar psychiatric risk factors (10,11). Impulsive subjects are also more likely to have suicidal ideation (12). Impulsivity is a fundamental component of theories of suicidal behaviors. Trait-dependent theories such as the stress-diathesis model of suicide (10) emphasize the importance of an interaction between negative life events or psychiatric states and underlying traits of impulsivity and suicidal feelings (e.g., hopelessness and suicidal ideation) to promote suicidal acts. State-dependent theories such as escape theory (13) emphasize that impulsivity is the product of the diminished capacity of the individual, or a “deconstructed state”, resulting in an increased suicide risk. Attempters with high impulsivity differed from attempters with low impulsivity, with the former showing evidence of more aggressive behaviors and a higher incidence of alcohol and drug abuse or dependence. In addition, adverse life events seem to play a role in precipitating suicidal behavior in impulsive individuals (14). Indeed, the relationship between impulsivity and suicide has been demonstrated in adolescent samples. For example, researchers have examined inpatient adolescents who had engaged in self-poisoning (versus those who did not and community controls) and determined that the overdose group was more impulsive than either of the control groups, even when they were being controlled for depressive symptoms (15). A recent study reported that alcohol/drug abuse is one of the most significant psychiatric risk factors associated with child and adolescent suicidal behavior (1). Substance abuse and dependence are associated with suicidal behaviors and this association may involve an interaction with impulsivity. Substance abuse is frequently related to an increased risk for expressing suicidal behaviors, which include suicidal ideation (16), suicide attempts (17), and suicide deaths (18). Substance abuse may impart greater suicide risk by increasing the impulsivity (17). In a review (19), researchers have provided a number of conclusions that supported the relationship between substance abuse and impulsivity: substance abusers were more impulsive on self-report and laboratory behavioral measures; impulsive groups had a higher incidence of substance abuse; impulsivity was both a risk factor for the development of substance abuse and a resulting consequence of substance abuse; impulsivity was a significant predictor of quitting drug treatment; and treatments for impulsivity improved outcome in substance abuse. The combination of suicidal behaviors, substance abuse, and impulsivity may produce synergistic effects (17).

A number of studies have reported an association between suicidal behavior (suicidal ideation and suicide attempt) in parents and suicide behavior (suicidal ideation and suicide attempt) in offspring and results have shown linkages between family histories of suicide and suicide attempt and increased risk of suicide behavior among family members (20-23). It has been ascertained that a responsibility to friends may be a reason for some individuals not to commit suicide (24). Some suicide attempters reported the loss of an important person in their life (25).

According to the literature, the comparison of impulsivity and its components (attentional, motor, and non-planning impulsiveness), substance abuse, and family/friends history of suicide attempt between suicide ideated and non-suicide ideated university students were examined in this study. In this research, it was hypothesized that family history of suicide attempt and loss of a significant person would be associated with an increased suicidal ideation among young adult university students.

## Materials and Methods

*Design:* The aim of this cross-sectional study was to compare the impulsivity, substance abuse, and family history of suicide attempt between suicide-ideated and non suicide-ideated university students. Data were analyzed using SPSS software (Version 16.0). For the description of data, mean and standard deviation were used. Multivariate analysis of variance was also performed to examine the hypothesis (α< 0.05).

*Sample:* The research population consisted of all the students of the University of Isfahan in the academic year of 2009-2010. Three hundred and forty students (136 boys and 204 girls based on the gender ratio in the population) were selected randomly through cluster sampling, of whom 53 participants were suicide-ideated and the rest were non suicide-ideated according to their answers to the research questionnaires. The rules of privacy of the subjects’ answers were confirmed in the questionnaires’ instructions.

## Instruments

*The Barratt Impulsiveness Scale, Version 11 (BIS-11):* The BIS-11 is a 30-item self-report questionnaire designed to assess general impulsiveness taking into account the multi-factorial nature of the construct. The structure of the instrument allows for the assessment of six first-order factors (attention, motor, self-control, cognitive complexity, perseverance, cognitive instability) and three second-order factors (attentional impulsiveness [attention and cognitive instability], motor impulsiveness [motor and perseverance], non-planning impulsiveness [self-control and cognitive complexity]). A total score is obtained by summing the first or second-order factors. The items are scored on a four-point scale (Rarely/Never [1], Occasionally [2], Often [3], Almost Always/Always [4]) (26). The internal consistency coefficient for the BIS-11 total score is ranged from 0.79 to 0.83 for separate populations of under-graduates, substance-abuse patients, general psychiatric patients, and prison inmates. The validity of the test is also significant as its factors were consistent with previous studies and factor intercorrelations were high (26). Cronbach’s alpha coefficients in Persian versions of Barratt Impulsivity Scale (BIS-11), Dickman Impulsivity Inventory (DII-2), Eysenck Impulsivity Questionnaire (EIQ-7), and Zuckerman Sensation Seeking Scale (SSS-5) in both normal and substance abuser groups ranged from 0.40 to 0.83. The subgroups of questionnaires in BIS-11 showed a significant correlation (r= 0.40). There was a significant correlation between impulsivity factors in other questionnaires and BIS-11 results. BIS-11 scores and impulsivity factors of other questionnaires in the substance abusers’ group was significantly (p<0.001) higher in comparison with normal subjects (27).

*The Suicide Ideation Questionnaire:* The Suicide Ideation Questionnaire (SIQ) that was used for the current study is the short version of the SIQ which has been shown to differentiate suicide ideators from non suicide ideators in several studies (28). It consists of four categories and requires the subjects to select the category that relates to their situation the most:

1. I have attempted suicide (to kill myself) in the past.

2. I have seriously considered committing suicide in the past to the extent I have made a plan on how I would do it but never followed through with the plan, or I have thoughts about harming myself that do not seem to go away.

3. The thought of committing suicide has crossed my mind, but I have never seriously considered it or made a plan in the past.

4. I have never thought about committing suicide.

Subjects who select categories one or two are classified as suicide-ideators and those who select categories three or four are classified as non suicide-ideators (28). The psychometric properties of the SIQ were acceptable according to the previous research (28). The SIQ was translated into Persian in parallel by two independent, native Iranian psychology professional translators, fluent in both English and Persian. Subsequently, two translators compared the Persian version and the original English version of the questionnaire. Pre-testing was completed with 30 subjects to evaluate the comprehension and readability of the questionnaires. Subjects were asked whether they encountered any difficulty in understanding each of the items. Subjects indicated they had no problems with the measures and understood the items. The content validity of the Persian version of SIQ was confirmed by five psychology faculty members. The concurrent validity of the Persian version of SIQ was obtained by correlating the score of this questionnaire with the Paykel’s Instrument for Measuring Suicidal Ideation and Attempts (29) according to their ability to differentiate suicide-ideators from non suicide-ideators in Iranian university student samples (phi coefficient= 0.88). Besides, Test-retest reliability of the Persian version of SIQ was reasonable (phi coefficient = 0.96).

*The demographic questionnaire:* In this study, a demographic questionnaire was used to assess the history of suicide attempts in subjects’ family/ friends, and the rate of their alcohol/drug abuse.

## Results

For hundred and twenty five students agreed to participate in this study. Three hundred and forty (80%) students completed and returned the questionnaires. Mean age was 21.86 (SD=2.03) years (Range: 18-30). Two hundred and four (60%) of the respondents were female, and one hundred and thirty five (40%) of them were male. The incidence of alcohol/drug abuse in suicide-ideators was higher in comparison with non suicide-ideators (26.6% vs. 7.5%). Furthermore, the incidence of suicide attempts in family (49% vs. 24% for survived attempts and 31.5% vs. 12% for attempts leading to death), and friends (38% vs. 17.5% for survived attempts and 27% vs. 9.5% for attempts leading to death) of suicide-ideators was higher compared with that of non suicide-ideators.

As shown in table 1, the mean scores of impulsivity and its components were higher in suicide-ideators compared with non suicide-ideators.

**Table 1:** Comparison of impulsivity and its components in suicide-ideators with non suicide-ideators

	**Group**	**Mean**	**Std. Dev.**
Impulsivity	Suicide-ideators Non suicide-ideators	66.06 61.42	10.41 9.23
Attentional impulsiveness	Suicide-ideators Non suicide-ideators	19.35 17.88	3.54 3.43
Motor impulsiveness	Suicide-ideators Non suicide-ideators	21.56 19.85	5.41 4.12
Non-planning impulsiveness	Suicide-ideators Non suicide-ideators	25.05 23.68	4.27 4.29

**Table 2:** Correlation coefficients among impulsivity and its components with age, gender, and marital status

	Age	Gender	Marital status
Impulsivity			
r	- .11	.10	.12
Sig.	.13	.22	.20
Attentional			
r	-.09	-.05	.06
Sig.	.20	.41	.22
Motor			
r	.05	-.10	-.08
Sig.	.29	.21	.31
Non-planning			
r	-.05	.07	.11
Sig.	.31	.51	.35

As shown in table 2, there were not any significant correlation between impulsivity and its components and demographic variables, namely age, gender, and marital status. For the comparison of suicide-ideated and non suicide-ideated groups in terms of impulsivity and its components (attentional, motor, and non-planning impulsiveness), a multivariate analysis of variance (MANOVA) was used to examine the multivariate effects of group membership on variables. The result from Box’s test of equality of covariance matrices showed that the observed covariance matrices of the dependent variables were equal across groups (F= 2.51, p< 0.20).

**Table 3:** The results from Wilk’s lambda test for examining the multivariate effects of group membership on impulsivity and its components

Effect	F	Hypothesis df	Error df	Sig.	Partial Eta Squared	Observed Power
Group	3.83	3	336	0.001	0.30	0.81

Table 3 shows that centroids of suicide-ideated and non suicide-ideated groups were significantly different regarding impulsivity, attentional, motor, and non-planning impulsiveness (F= 3.83, p< 0.001). It means that these two groups differed in at least one of the mentioned variables, namely impulsivity, attentional, motor, and non-planning impulsiveness. According to the partial eta-quared coefficient, 30 percent of group membership variance was attributed to impulsivity. The observed power (0.81) shows the sufficiency of the sample.

A univariate analysis of variance (ANOVA) was used to determine the univariate effects of group membership on each of the variables (Table 3).

**Table 4:** The results from univariate analysis of variance for examining the univariate effects of group membership on impulsivity and its components

Dependent Variable	Type III Sum of Squares	df	Mean Square	F	Sig.
Impulsivity	964.224	1	964.224	10.853	.001
Attentional	96.806	1	96.806	8.123	.005
Motor	144.986	1	144.986	7.677	.006
Non-planning	84.125	1	84.125	4.603	.033

Table 4 shows that there were significant differences in impulsivity (p<0.001), attentional (p< 0.005), motor (p< 0.006), and non-planning (p< 0.033) impulsiveness between suicide-ideated and non suicide-ideated groups.

## Discussion

This research aimed to compare the impulsivity, substance abuse, and family/ friends history of suicide attempts between suicide-ideated and non suicide-ideated university students. This study showed that there were significant differences between suicide-ideated and non suicide-ideated groups in impulsivity and its components (attentional, non-planning, and motor impulsiveness). These results are consistent with previous manuscripts (3,4,6-12). As noted before, impulsiveness and aggressiveness traits in adolescents and young adults are related to psychosocial adjustment problems and even suicide risk. Most theories of suicidal behavior have indicated that impulsivity and self-harm are directly related to each other. In other words, “spur of the moment” behavior is responsible for higher rates of suicidal behavior among impulsive people. It means that individuals who complete or attempt suicide may show impulsiveness more frequently than demographically similar non-suicidal individuals and impulsive subjects are probably more likely to have suicidal ideation. The findings of this research about the different quantity of substance abuse between suicide-ideated and non suicide-ideated students are consistent with previous findings (1,14-19). It means that suicide-ideators with high impulsivity may differ from non suicide-ideators with low impulsivity, with a higher incidence of alcohol and drug abuse or dependence. In other words, according to the previous research, impulsive suicides are more likely characterized by a higher prevalence of lifetime substance abuse/ dependence. As noted before, substance abuse is frequently related to an increased risk for expressing suicidal behavior, which includes suicidal ideation, suicide attempts, and suicide deaths. Thus, this study suggests that substance abuse is probably associated with suicidal ideation and this association may involve an interaction with impulsivity.

Another finding of this study was the higher incidence of suicide attempts in the family/friends of suicide-ideated in comparison with non suicide-ideated students. According to this finding and in line with previous research (20-25), the hypothesized linkages between family histories of suicide and suicide attempt and increased risk of suicide ideation among family members have been confirmed by the results of this study. Therefore, this study suggests that the loss of an important person (a family member or a friend) in life would be an important predictor of suicidal ideation in university students. Besides, this study provides an initial evidence of familial linkages of suicide ideation and suggests that the loss of an important person in life would be an important predictor of suicide ideation in university students.

## Conclusions

In conclusion, the results of this study were more consistent with trait-dependent theories such as the stress-diathesis model of suicide, which emphasizes the importance of an interaction between negative life events or psychiatric states and underlying traits of impulsivity and suicidal feelings (e.g., hopelessness and suicidal ideation) to promote suicidal acts. Suicide is a multifaceted illness with numerous interacting causes and risk factors that frequently co-occur. More researches are needed to provide a comprehensive theoretical explanation for the interrelationships among impulsivity, suicidal ideation, substance abuse, familial transmission of suicidal behavior, and loss of an important person.

## Authors' Contributions

First author conceived and designed the evaluation and helped to draft the Manuscript, designed the evaluation and performed the statistical analysis. Other authors participated in revising the manuscript.

## Notes

**Declaration of Interest:** None.

**Clinical Trial Registration: **URL: http://www.Irct.ir/Unique identifier: IRCT201104251457N8
